# Schistosomiasis mansoni incidence data in Rwanda can improve prevalence assessments, by providing high-resolution hotspot and risk factors identification

**DOI:** 10.1186/s12889-017-4816-4

**Published:** 2017-10-25

**Authors:** E. Nyandwi, A. Veldkamp, S. Amer, C. Karema, I. Umulisa

**Affiliations:** 1 Faculty of Geo-Information Science and Earth Observation (ITC), University of Twente, P. O. Box 217, 7500 AE Enschede, the Netherlands; 2 Geographic Information Systems and Remote Sensing Centre of University of Rwanda, P.O Box 212, Huye, Rwanda; 3 Rwanda Biomedical Centre/ Malaria and Other Parasitic Diseases Division, P. O. Box 2514, Kigali, Rwanda; 4 Swiss Tropical and Public Health Institute, Socinstrasse 57, P.O. Box, CH-4002 Basel, Switzerland; 5 Universität Basel, Petersplatz 1, CH-4003 Basel, Switzerland

**Keywords:** Schistosomiasis mansoni, Incidence rates, Risk factors, Spatial scale, Empirical model

## Abstract

**Background:**

Schistosomiasis mansoni constitutes a significant public health problem in Rwanda. The nationwide prevalence mapping conducted in 2007–2008 revealed that prevalence per district ranges from 0 to 69.5% among school children. In response, mass drug administration campaigns were initiated. However, a few years later some additional small-scale studies revealed the existence of areas of high transmission in districts formerly classified as low endemic suggesting the need for a more accurate methodology for identification of hotspots. This study investigated if confirmed cases of schistosomiasis recorded at health facility level can be used to, next to existing prevalence data, detect geographically more accurate hotspots of the disease and its associated risk factors.

**Methods:**

A GIS-based spatial and statistical analysis was carried out. Confirmed cases, recorded at primary health facilities level, were combined with demographic data to calculate incidence rates for each of 367 health facility service area. Empirical Bayesian smoothing was used to deal with rate instability. Incidence rates were compared with prevalence data to identify their level of agreement. Spatial autocorrelation of the incidence rates was analyzed using Moran’s Index, to check if spatial clustering occurs. Finally, the spatial relationship between schistosomiasis distribution and potential risk factors was assessed using multiple regression.

**Results:**

Incidence rates for 2007–2008 were highly correlated with prevalence values (R^2^ = 0.79), indicating that in the case of Rwanda incidence data can be used as a proxy for prevalence data. We observed a focal distribution of schistosomiasis with a significant spatial autocorrelation (Moran’s *I* > 0: 0,05–0.20 and *p* ≤ 0,05), indicating the occurrence of hotspots. Regarding risk factors, it was identified that the spatial pattern of schistosomiasis is significantly associated with wetland conditions and rice cultivation.

**Conclusion:**

In Rwanda the high density of health facilities and the standardized microscopic laboratory diagnostic allow the derived data to be used to complement prevalence studies to identify hotspots of schistosomiasis and its associated risk factors. This type of information, in turn, can support disease control interventions and monitoring.

## Background

Schistosomiasis remains one of the most prevalent water-based diseases in the tropics. Regarding the impact, it is considered the second most important parasitic disease after malaria in many countries in sub-Saharan Africa [1, 2]. In Rwanda, schistosomiasis mansoni, (written as “*S. mansoni*” in this paper) with district level prevalence ranging from 0 to as much as 69.5% among school children constitutes a significant public health problem. The overall country prevalence in 2007–2008 was 2.7%.

The Neglected Tropical Disease (NTD) control program was established in 2007 by the Ministry of Health to fight against five NTDs which pose a significant public health problem. This program included the Schistosomiasis Control Initiative, which was implemented using the nationwide school-based prevalence map of 2007–2008 as a guideline. The prevalence recorded at 2 to 4 surveyed schools per district was averaged to estimate prevalence at the district level (nationwide 136 schools were surveyed). The Mass Drug Administration (MDA) for *S. mansoni* targeted children in areas with a prevalence of at least 10% and included adults where prevalence exceeded 30% [3].

However, two years later, some health facility located in districts classified as having a low prevalence, recorded higher frequencies of *S. mansoni* infection. In addition, some small-scale prevalence surveys revealed the existence of localized geographic areas with higher prevalence (up to 77.9%) than previously reported [4, 5]. One such study identified a ‘new’ area with the high rates of schistosomiasis [6], which was not detected before by the national prevalence study. The latter confirms the general recommendation of prevalence based studies in Rwanda or elsewhere in Africa [7, 8] that more detailed information is required to address the often highly focalized spatial pattern of schistosomiasi*s* hotspots. The same studies also recommend to include other high-risk community groups (e.g.: women of children bearing age, rice farmers, fisherman) in future investigations.

**Fig. 1:**
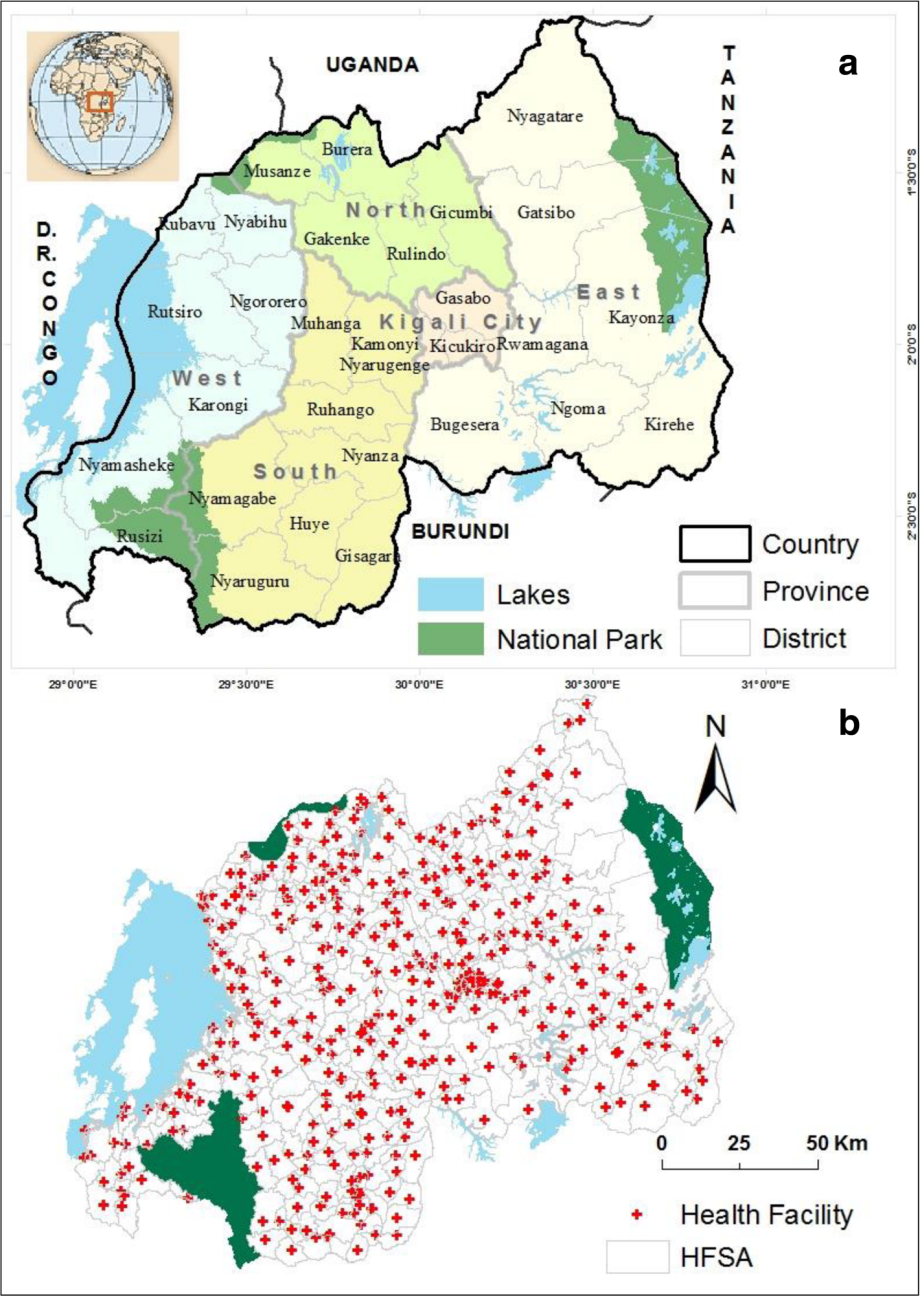
Map of Rwanda. The 30 administrative districts with Province boundary shown in different colors (**a**) Primary health facility (HF) location with their respective service area (HFSA) boundary (**b**). There are few health facilities in a strip of relatively larger HFSAs in East of the country recently and lowly populated by cattle farmers settled in grouped settlement far from National park boundary and former hunting domain

To achieve this, the nationwide school-based prevalence surveys would need to include a very large number of schools and other high-risk community groups to provide sufficient information to identify and delineate hotspots. In Rwanda, districts are relatively large administrative units (see Fig. 1a). To overcome the low granularity of the current mapping method [9], the prevalence surveys would require large numbers of sample locations and becomes very expensive and harder to execute. Consequently, there is a clear need to explore the value of other sources of health data and alternative mapping approaches to complement prevalence inventories and support planning and implementation of *S. mansoni* control programs. For other water based diseases, incidence data from routine health statistics have been successfully used to complement prevalence studies and identify the spatial distribution of a given disease [10–12]. Given the availability of good quality spatially structured and systematically collected data at the health facility level, use of such incidence data seems feasible in Rwanda.

Since incidence and prevalence have a direct relationship (prevalence = incidence rate x average duration of disease), a logical first step is to explore to what extent both have a similar spatial distribution. If there is a significant level of the agreement, it can be useful to use the much higher resolution incidence data for improved mapping of hotspots, and so more efficiently guide control interventions to high-risk locations.

From previous studies, it is known that the transmission of *S. mansoni* follows complex pathways depending strongly on the dynamics of the local biophysical and socio-economic context. Socio-economic and biophysical factors together influence when and where humans are in contact with water potentially contaminated with freshwater snails [13]. In the Rwandan situation, new infrastructural developments such as dams and irrigation scheme expansion could very well contribute to the spread of *S. mansoni* to previously non-endemic areas [14]. The association with irrigation projects is well scrutinized by Steinmann et al. [15] in a systematic review of the relation between schistosomiasis occurrence and irrigated areas in some African countries. Specific socio-economic conditions such as educational attainment, access to improved water sources, and proper sanitation, are also known to be related to *S. mansoni* infection in endemic areas [16, 17].

This study aims to investigate if schistosomiasis incidence data recorded at health facility level can provide, next to existing prevalence data, additional insights into the spatial pattern of schistosomiasis occurrence. Given the fine-grained spatial resolution of health facility service areas in Rwanda, this could allow more detailed spatial hotspot detection of the disease and its associated risk factors.

## Methods

### Study area

Rwanda is a relatively small landlocked country of 26,338 km^2^ in the Great Lakes region of Central-Eastern Africa with a climate characterized by two rainy and two dry seasons. Administratively, Rwanda is divided into five provinces, 30 districts and 416 sectors [18]. The sectors together make up 367 health facility service areas (HFSAs), as shown in Fig. 1b. Around 11 million people inhabit the country [19], of which 83.5% live in the countryside mainly engaged in small-scale farming. Because of shortages of agricultural land, wetland conversion is one of the ongoing activities for rural development [20].

Over time, Rwanda’s health sector has experienced a profound evolution from traditional healing methods to faith-based health care during the colonial period, then centralized, and free provision of health services up to the early 1990’s, to the current decentralized health care delivery system. The decentralization reinforces community participation in the management and financing of health services. The current public health care delivery system is a hierarchically organized three-tier system providing primary, secondary and tertiary health care. Primary health care is provided at sector level via one or more health facilities, secondary care is provided in the districts by a district hospital, and referral hospitals provide tertiary care at the regional scale. Each health facility reports to the district health office, which is responsible for the health facilities and services provided to the population of the district. Community Health Workers (CHWs)function as an effective link between communities and public health care initiatives (Health Policy of Ministry of Health elaborated in 2005).

### Data collection and quality checking

#### Administrative boundaries and spatial delineation of health facility service areas

The geographic location of health facilities depicting the 2007 situation, were provided by the Rwanda Biomedical Centre - Malaria and Other Parasitic Diseases Division (RBC/M&OPD). For this study, the health facility data were updated in May–June 2013 via intensive consultation of District Land Officers, Surveyors, and GIS Technicians from each administrative district. Furthermore, we spatially demarcated the HFSA using the ‘cost allocation’ spatial analyst tool of ArcGIS. Then, the delineated areas were further adjusted to the administrative units considering the population size, physical and managerial boundary as planned for Community-Based Health Insurances (CBHI) scheme management (In Ministerial Instruction Nr /Min/2012 on District - Health -Guidelines, 2012). The administrative boundaries of the country at different levels were obtained from the National Institute of Statistics of Rwanda. The general information data sets such as lakes, islands, parks, and roads, have been acquired from the Centre for GIS and Remote Sensing of the University of Rwanda (CGIS-UR).

#### Schistosomiasis mansoni incidence data

The number of confirmed cases of *S. mansoni* for the years 2007–2012 were provided for this study by the RBC/M&OPD. The same department also provided point locations and prevalence data obtained at the schools included in the nationwide school-based prevalence survey of 2007–2008. Another prevalence map produced using supra-national data, and regional simulation was also available from the World Health Organization [21]. The quality of recorded schistosomiasis infection cases at HFSA level is sound for six reasons. First, *S. mansoni* infection is diagnosed via microscopic identification of eggs in stool samples in the laboratory of the health facility. Second, accessibility is unproblematic as patients can reach a health facility within a walking distance of at most 5 km. Third, Community-Based Health Insurance (CBHI) makes that appropriate health care is affordable for everyone (patients pay only 10% of the total cost of service and medication). Fourth, community health workers actively stimulate patients to visit the health facility in case of suspected health problems. Fifth, since there is no traditional medicine used for Schistosomiasis in Rwanda, patients with symptoms will go for treatment at the health facility. Sixth, the Rwandan Health Management Information System (R-HMIS), routinely and systematically collects health records from individual health facilities using a web-based software platform (DHIS2) whereby each health facility enters their monthly health records directly into the national database [22].

#### Socio–economic and biophysical covariates

Demographic data were extracted from the 2002 and 2012 Population, and Housing Census published by the National Institute of Statistics of Rwanda (NISR) and for the years in between the estimated population number and distribution were extracted from estimation and projections also reported by NISR. The socio-economic factors such as school attendance levels, access to improved water sources, proper sanitation at district and HFSA level were available from census reports.

*S. mansoni* transmission is determined and accelerated by interactions of various factors spatially restricted to freshwater bodies inhabited by particular host snails [23]. Earlier studies in East African countries identified numerous biophysical and socio-economic conditions related to schistosomiasis infection risk [1]. In addition to socio-economic factors, biophysical factors need to be considered as well. Wetland agro-ecosystems related factors (namely wetland proportion, rice cropped areas, wetland/water body adjacency) were also collected. Likewise, topographic and climatic factors were used as potential risk factors for this study. Data acquisition and pre-processing methods to generate raster data for the risk factors are detailed in previous research on Rwandan wetlands characterization and their climate sensitivity [24]. Soil parameters (pH, clay, and sand content percentage) were extracted from the soil geo-database of Rwanda generated from a semi-detailed soil survey consisting of 1833 soil profiles spread over the country. The geostatistical interpolation of soil properties was done using landform data at a scale of 1: 250,000 and 1: 50,000 [25]. Mean values at HFSA level were extracted from original risk factors raster data using zonal statistics tools of ArcGIS 10.2.2. District factor values are the average of values of HFSAs within a district.

### Comparison of spatial distribution of *S. mansoni* with incidence and prevalence datasets

Incidence and prevalence are both measurements of disease frequency. Incidence estimates how often disease occurs in space and time (a measure of disease risk). Prevalence evaluates how much the disease is spread in a given population (a measure of disease burden) at a given moment. Since both are related (prevalence = incidence rates x average duration of disease), high prevalence areas may correspond with high incidence rates for a disease such as *S. mansoni.* The 2008 prevalence at each of the 136 surveyed schools is directly compared with incidence rates at HFSA level for the same year and location. Prevalence and incidence data were also mapped at the district level to enable visual comparison.

### Detection and visualization of the spatial pattern of *S. mansoni*

Appropriate spatial and statistical approaches for detecting spatial clustering and relationships with risk factors are provided by current advances in spatial epidemiology [26, 27].

#### Cases of *S. mansoni* per population at risk

The number of confirmed cases of *S. mansoni* per HFSA per month were summed per year and further aggregated to the district level. The generated annual *S. mansoni* incidence cases and demographic data were joined using Excel and then linked to the HFSA and district spatial data. The incidence rate is the number of *S. mansoni* cases per district (*n* = 30) or HFSA (*n* = 367) divided by the population of that district or HFSA, as shown by the equation below:

$$ \mathrm{Di}=\left(\mathrm{In}/\mathrm{Pt}\right)\ast 100\ 000 $$

Where ***Di*** is the *S. mansoni* incidence rate, ***In*** is the total number of new cases in 12 months of a year per district/HCSA, and ***Pt*** is the total population of that year for that entity.

To make the rates more intuitive, they were multiplied by 100,000 to obtain incidence rates reported per one hundred thousand persons [28]. The raw rates from sparsely populated HFSAs were replaced by weighted averages using Empirical Bayesian Smoothing (EBS) [29]. EBS computes raw rates and produces three weighted rates using the global, mean and local average. The smoothing was done using the SpaceStat software [30].

For visualization, incidence rates were classified into four classes using the Jenks classification. The classes defined in this way were harmonized to allow for inter-annual comparison and comparison with prevalence maps at the district level. Furthermore, the average incidence rates for 2007 and 2008 at HFSA level were standardized to vary from 0 to 1 (from non-endemic to hyperendemic areas) and superimposed with the points map of the 136 schools surveyed during the nationwide mapping of 2007–2008. A scatterplot was then generated to display the relationship between incidence rates at HFSA level and prevalence per school.

#### Analyzing pattern with Moran’s index statistic

Spatial autocorrelation was computed to ascertain the correlation between neighboring incidence rates of *S. mansoni* and the level of spatial clustering within the study area [31]. The Moran’s Index statistic, similar to the Pearson correlation [32], is widely used for this and was calculated as:

$$ I=\frac{N}{S_O}{\sum}_i{\sum}_j{w}_{ij}\frac{\left({x}_i-u\right)\left({x}_j-u\right)}{\sum_i{\left({x}_i-u\right)}^2}, $$

Where ***N*** is the number of districts/HFSA; ***W***_***ij***_ is the element in the spatial weights matrix corresponding to the observation pair ***i, j.*** Also, ***x***_***i***_ and ***j***_***i***_ are observation for areas ***i*** and ***j*** with mean ***u***. And

$$ {S}_O={\sum}_i{\sum}_j{w}_{ij} $$

Since the weights are row-standardized ∑***w***_***ij***_ = 1, the first step in the spatial autocorrelation analysis is to construct a spatial weight matrix that contains information about the neighborhood structure for each location. Adjacency is defined as the immediately neighboring district/HFSA, including the district/HFSA itself [33]. Non-neighboring units have a weight of zero.

#### Mapping clusters

The Local *G*_*i*_ *(d) statistic was selected to test the statistical significance of local clusters and to determine the spatial extent of these clusters [34, 35]. The Local *G*_*i*_ *(d) statistic is useful for identifying individual members of local clusters by determining the spatial dependence and neighboring observations [26, 36]. It can be written as follows:

$$ {G}_i^{\ast }(d)=\frac{\varSigma_j{w}_{ij}(d){x}_j-{W}_i\overline{x}}{s\sqrt{\frac{\left({nS}_{1i}-{W}_i^2\right)}{\left(n-1\right)}}},\kern0.5em for\kern0.5em all\kern0.5em j $$

Where *x* is a measure of incidence rate of *S. mansoni* within a given district/HFSA polygon; *W*_*ij*_ is a spatial weight that defines neighboring district/HFSA *j* to *i*; *W*_*i*_ is the sum of the weight *W*_*ij*_ *,*

$$ \overline{x}=\frac{1}{n}{\sum}_j{x}_j\kern1em {s}_{1i}={\sum}_j{w}_{ij}^2,\kern1em {s}^2=\frac{1}{n}{\sum}_j{x}_j^2-{\overline{x}}^2. $$

Developing the spatial weight *W*_*ij*_ is the first step to calculating Gi* (d). The spatial weight matrix includes *W*_*ij*_ = 1. and in this study, the adjacency has been defined in ArcGIS - proximity analysis based on polygons that share common boundaries and vertices [37].

With Local *G*_*i*_ *(d) statistic clusters with a 95% significance level from a two-tailed normal distribution indicate significant spatial clustering, but only positively significant clusters are mapped.

### Empirical modeling of *S. mansoni* with potentially associated factors

Input data sets were standardized and prepared at HFSA level using standard functionality of ArcGIS. The attributes of the spatial data were exported to IBM SPSS Statistics, version 20 which was used for all statistical analyses. Before the use of the variables to train an exploratory empirical model, exploratory data analysis was done. Considering the patterns of data distribution, using normal distribution curves and frequency distributions; a log-normal distribution transformation was done for some of the input datasets [38]. Then, with normally and ln-transformed variables, Pearson’s rank correlation coefficient and the test of co-linearity using pairwise scatter plots was done. Sixty percent of the data was randomly sampled for the model, to avoid the spatial autocorrelation in our empirical regression and to prevent a fixed spatial pattern. With the spatial autocorrelation, the model becomes insensitive to changes in the spatial patterns and over fitted.

The incidence of *S. mansoni*, as the dependent variable, was related to all potential risk factors. Risk factors used in the statistical analysis were grouped into five. The first group is made up of physical variables, sub-categorized as soil properties (pH, sand percentage, and clay percentage) and terrain derivatives (elevation, slope, and terrain shape index). The second group represents ecological variables (wetland proportion, the total area of rice cropping schemes, water/lake adjacency). The third group consists of two climatic variables (total annual amount of rainfall and average annual temperature). The fourth group consists of four demographic variables (number of households, population density, and percentage of rural and urban residents). The last group represents socio-hygienic conditions, namely: level of education, the source of water and sanitation situation.

A stepwise linear regression was conducted, considering *P* > 0.1 as the removal criterion and *P ≤* 0.05 as the entry criterion. This quantifies the strength of the relationship between schistosomiasis cases and the significant covariate(s). The standardized coefficients were used to compare the effects of each independent variable on the dependent variable [39]. To determine how well the regression model fitted the data, we used the R-square and the Standard Errors of the Regression (S). We considered the S to represent the average distance that the observed values deviate from the regression line. The S must be ≤2.5 to produce a sufficiently narrow 95% prediction interval [40]. Also, we used both residuals and residual plots to analyze drift and variance of the values [41], for evaluating the appropriateness of the model.

## Results

### Comparing prevalence and incidence data

**Fig. 2:**
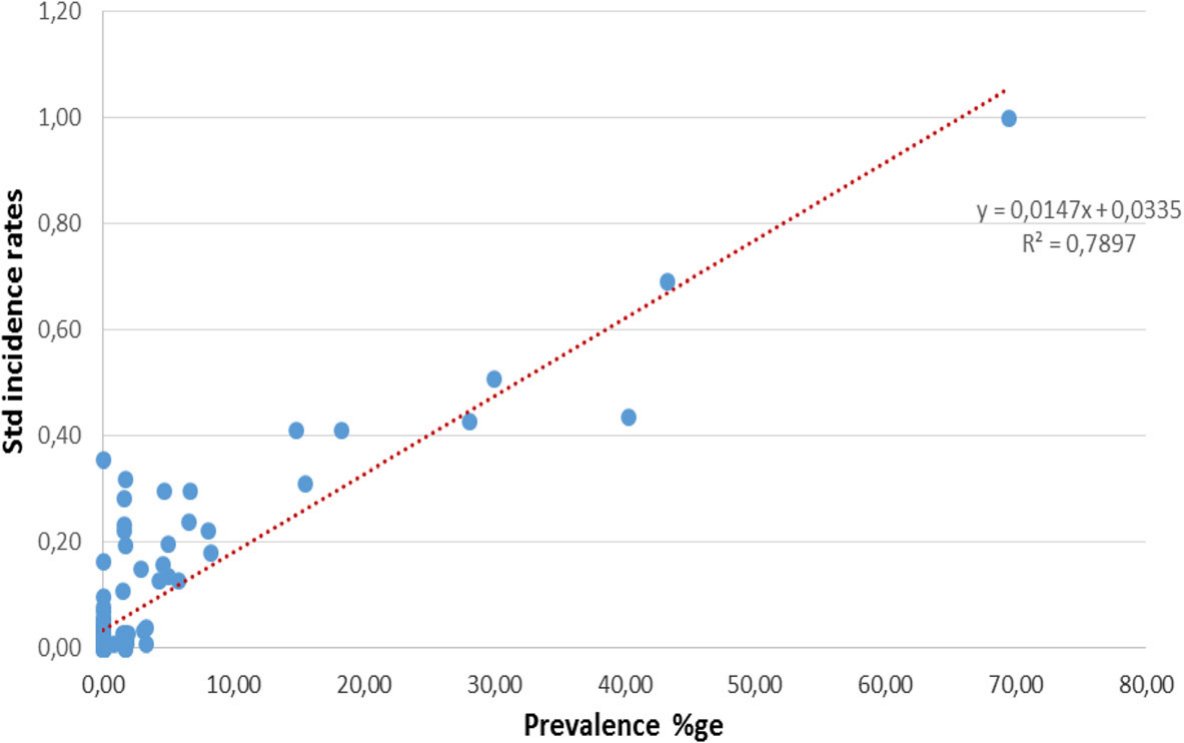
Scatter plot and trend line between Prevalence and Incidence values. The prevalence proportion measured at each of 136 surveyed schools linked to their location; and corresponding standardized incidence rates were extracted for the same places

To check if indeed there is a significant relationship between prevalence and incidence data, we compared standardized incidence rates of 2007–2008 at HFSA level with prevalence data obtained at the 136 school sampled during the 2007–2008 nationwide survey. In Fig. 2, we can observe a very strong relationship between the two data sources with a coefficient of determination of 0.79.

This strong relationship indicates that we can use routinely recorded cases of *S. mansoni* at primary health facility level to supplement prevalence data. The readily available incidence rates can be utilized as a proxy for prevalence. This information can be sourced on a monthly basis for every health facility for more than one decade.

### Spatial distribution of *S. mansoni* in Rwanda

**Fig. 3:**
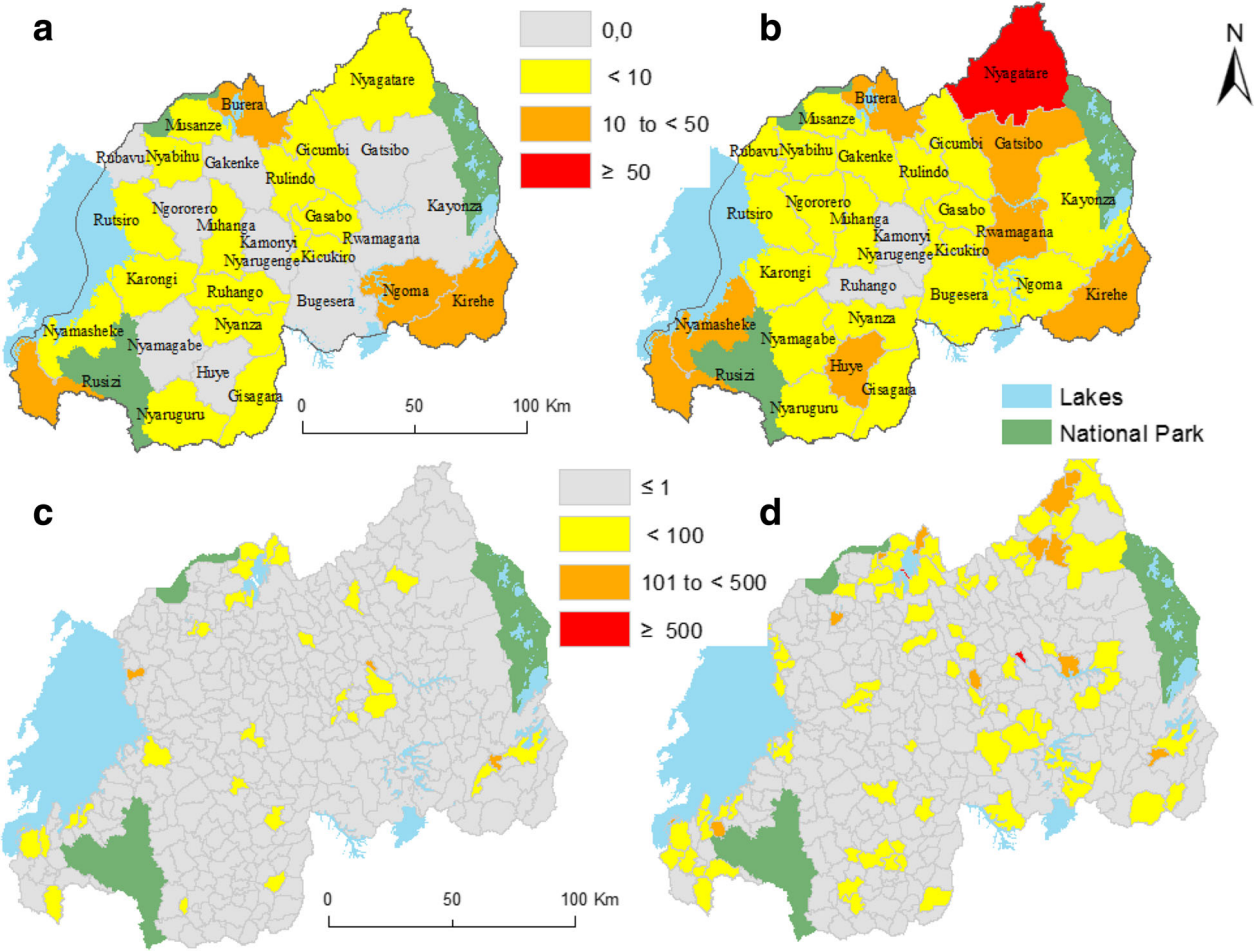
Incidence rates (per 100,000 persons) of *S. mansoni;* at the district level for 2007 (**a**) and 2008 (**b**) and EBS incidence rates at HFSA level for 2007 (**c**) and 2008 (**d**)

A total number of 1221 *S. mansoni* cases were reported in Rwanda for the years of 2007 and 2008. Annual incidence rates for 30 districts were calculated (Table 1). Nyagatare district had the highest rate of 57 cases per 100,000 persons in 2008. Then EBS incidence rates for 367 HFSAs were also calculated for the year 2007 and 2008. As visualized in Fig. 3, a lot of HFSAs, as well as some districts, have zero or very low numbers of *S. mansoni* cases per year. Visually, there also are districts with high rates (respectively Nyagatare, Kirehe, Ngoma, Rusizi, and Burera); districts with zero rates (10 Districts in 2007 and only 2 in 2008) and districts with very low incidence rates. At HFSA level, the spatial pattern of *S. mansoni* is much more distinct, showing considerable differences within a district.

**Table 1:** Incidence rates (per 100.000 persons) of *S. mansoni* in Rwanda for specific years (2007, 2008 and cumulative incidence (2007–2008)

District Name	Year		
	2007	2008	2007–2008
Nyarugenge	2,33	2,67	3,82
Gasabo	5,39	6,42	8,40
Kicukiro	0,41	1,17	0,78
Nyanza	0,38	7,05	3,71
Gisagara	1,05	1,37	1,72
Nyaruguru	0,39	1,51	1,34
Huye	0	11,43	5,71
Nyamagabe	0	0,64	0,32
Ruhango	0,72	0	0,71
Muhanga	0,66	0,98	1,31
Kamonyi	0	0	0
Karongi	1,66	0,33	1,96
Rutsiro	8,64	7,13	12,22
Rubavu	0	0,58	0,29
Nyabihu	3,92	6,7	7,05
Ngororero	0	4,2	1,94
Rusizi	17,91	21,66	28,42
Nyamasheke	4,57	15,46	12,37
Rulindo	0,75	1,47	1,47
Gakenke	0	1,81	0,91
Musanze	3	7,96	7,08
Burera	16,45	47,6	40,02
Gicumbi	2,65	8,4	6,83
Rwamagana	0	18,83	9,60
Nyagatare	1,57	57,07	30,04
Gatsibo	0	10,35	5,18
Kayonza	0	1,13	0,76
Kirehe	35,25	16,89	42,40
Ngoma	10,98	2,13	11,72
Bugesera	0	1,28	0,64

### Spatial autocorrelation of *S. mansoni* rates

The results of spatial autocorrelation of neighboring values aggregated at HFSA and district level are summarized in Table 2.

**Table 2:** Test of spatial autocorrelation of *S. mansoni* rates computed for cumulative incidence (2007–2008) and the particular years (2007 and 2008)

Year	District level		HFSA level	
	Moran’I (*p* value)	Z (I)	Moran’I (p value)	Z (I)
2007–2008	0.13 (<0.001)	5.03	0.14 (<0.001)	5.03
2007	0.24 (0.05)	1.89	0,12 (<0.001)	4.36
2008	0.12 (0.29)	1.10	0.11(<0.001)	3.65

The results were statistically significant at the district level and strongly significant at HFSA level (*p* < 0.05 and z-score greater than 1.96). The statistically significant values indicate that the distribution *S. mansoni* is spatially heterogeneous in Rwanda and that heterogeneity is more explicit at HFSA level.

### Measures of spatial clustering (hotspots) of *S. mansoni* rates

**Fig. 4:**
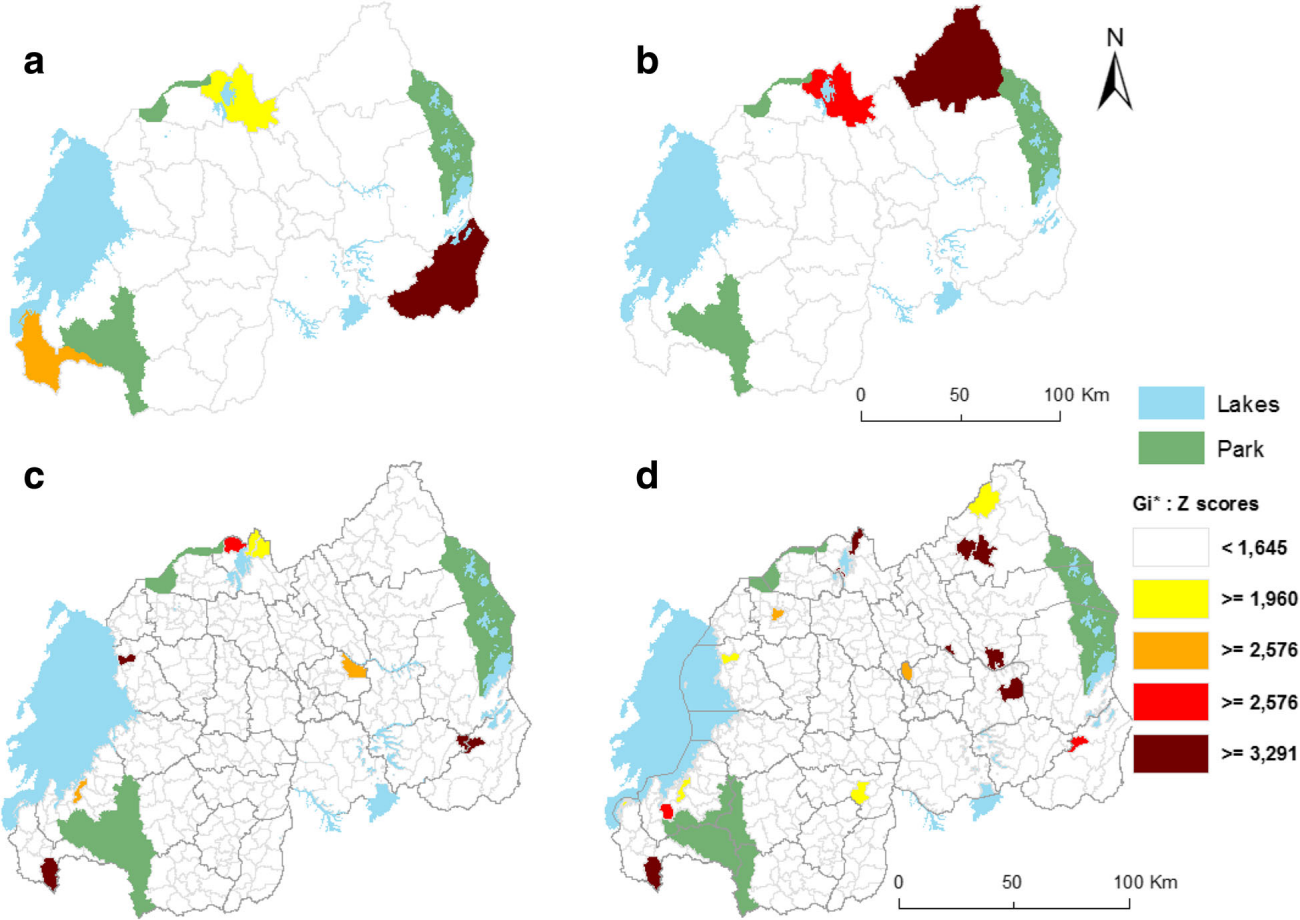
Spatial clusters of *S. mansoni* incidence rates. At District level in 2007 (**a**), in 2008 (**b**) and at HFSA level in 2007 (**c**), in 2008 (**d**). These maps show five levels of statistical significance of Z-score values: Not important spot, represented by white color has a value <1.645. Hot Spot with 90% confidence (yellow color) ≥1.645; follows ≥1.960 for Hot Spot with 95% Confidence (gold color); ≥ 2.576 Hot Spot with 99% Confidence (red color) and ≥3.291 Hot Spot with 99,9% Confidence (dark red color)

The identified statistically significant hotspot areas from the Local Gi* (d) test of *S. mansoni* rates for the year 2007 and 2008 are visualized in Fig. 4.

The outcomes from spatial clustering analysis computed with Local Gi* (d) statistic at district and HFSA levels are categorized as clusters (z-score ≥ 1.96) or non-clusters (z-scores <1.96), at different significance level.

### *S. mansoni* Spatial distribution in relation to environmental factors

Some risk factors have a significant relationship with *S. mansoni* incidence. The sand percentage (of the soil) and elevation are negatively correlated with *S. mansoni* incidence rates, while ln-terrain shape index (TSI), temperature, rain, and rice cropped area in wetlands is positively correlated (see Table 3). Using R-squared and Standard error of the estimate, the risk factors included in the empirical model explain quite a lot regarding the spatial distribution of *S. mansoni* with a distinct effect of spatial scale. More than 47% of the distribution (with S of 0.926) at detailed HFSA level and 60% (with S of 0.366) at the larger District level.

**Table 3:** Multiple regression outputs for the relationship between environmental factors and *S. mansoni* at HFSA and District level

Parameters	B Coeff.	Standard Error	Beta Coeff.	t-value	*p*-value
HFSA level model					
Intercept	−0.697	0.238	–	−2.931	0.004
Sand percentage	−0.004	0.001	−0.366	−3.816	0.000
Rice cropped area	0.001	0.000	0.256	2.692	0.009
log-TSI	0.131	0.041	0.298	3.187	0.002
Rain	0.001	0.000	0.281	2.585	0.012
Temperature	0.028	0.010	0.296	2.832	0.006
District level model					
Intercept	1.999	0.906	–	2.207	0.036
Sand percentage	−0.032	0.010	−0.447	−3.216	0.003
Elevation	−0.002	0.000	−0.661	−4.373	0.000
Rain	0.002	0.000	0.657	4.336	0.000

None of the two models included socio-hygienic variables such as educational attainment, the source of water or sanitation conditions. Even with the univariate test by Pearson’s rank correlation (not reported here), none of the hygienic and socio-demographic factors had a significant association with *S. mansoni* incidence.

## Discussion

This study identifies and visualizes the spatial variability of *S. mansoni* at two levels of spatial resolution using routinely collected health records as a basis. The incidence rates generated at HFSA levels were EBS smoothed, and the global mean was able to assign new rates, as recommended for disease mapping at a high spatial resolution [42] and in line with the considerable geographic concentration of *S. mansoni* in Rwanda [4]. The strong correlation between neighboring values at small scale was supported by the Global Moran’s index. The spatial clustering test using Local *G*_*i*_ *(d) statistic also shows the non-random spatial distribution of *S. mansoni*.

### Routine health records provide valuable information for spatial pattern detection of *S. mansoni*

Disease maps depicting the spatial pattern of *S. mansoni* are essential for guiding control program activities. However, the added value of disease maps much depends on their spatial resolution, and on the underlying data used to establish them [43]. In this section, we first compare the incidence rates based maps at district and HFSA levels. The second comparison is between incidence rates based mapping and prevalence based mapping.

**Fig. 5:**
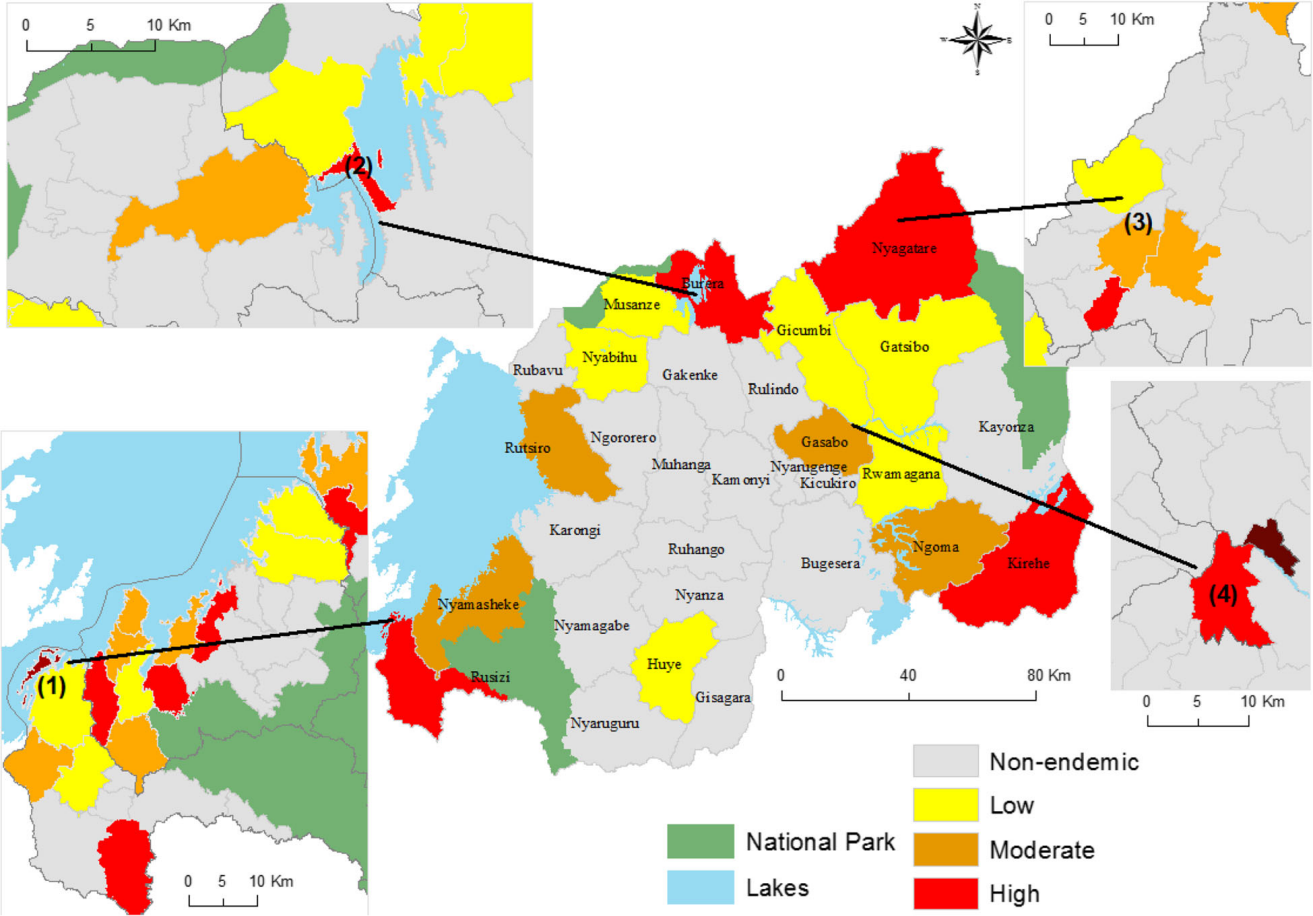
Spatial scale sensitivity of *S. mansoni* incidence rates. The large map in the centre shows incidence rates (2007–2008) per district. This is then compared with rates displayed at HFSA level using known hotspots areas as a reference

Figure 5 illustrates how spatial scale influences the detection of disease hotspots. The center map of Fig. 5 depicts incidence rates at the district level for 2007–2008, while the four smaller maps show the same information but now at the HFSA level. The HFSA level maps clearly show the highly focalized nature of *S. mansoni* hotspots. Representation at the district level, on the other hand, results in an overestimation of areas of high transmission as well as in non-identification of hotspots in districts with generally low incidence rates. The four identified hotspots areas at HFSA level (see Fig. 5) have also been identified by previous studies. The first and second hotspot areas are historically endemic zones of *S. mansoni*. Recently, Ruberanziza et al. [4] reported Nkombo Island (hotspot 1), as the most important *S. mansoni* focus in Rwanda. Ntaruka HFSA (hotspot 2) between Burera and Ruhondo lakes, was previously also identified as a high transmission area by several cross-sectional surveys [5, 44, 45]. Hotspot 3 in Nyagatare and hotspot 4 in Gasabo district have not been documented before.

**Fig. 6:**
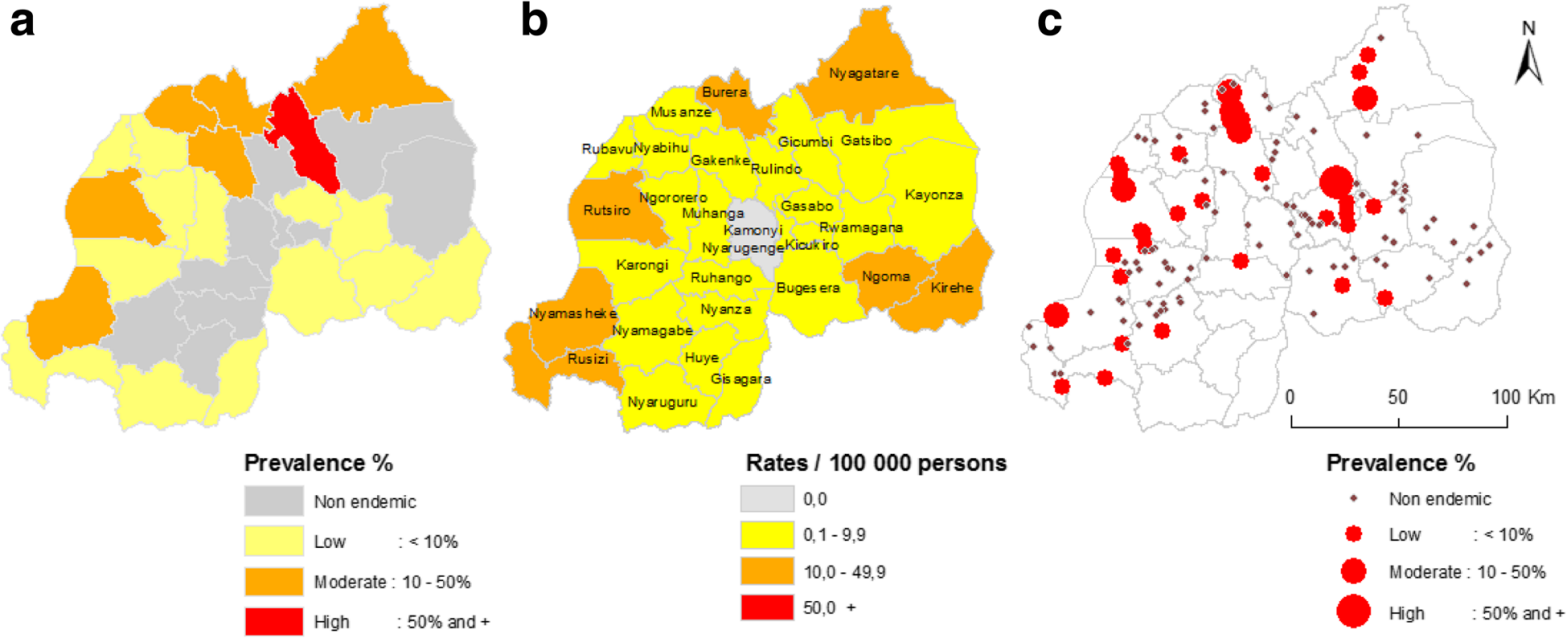
The spatial pattern of *S. mansoni* at the district level. The first prevalence map of 2008 was produced by WHO using global data and spatial simulation (**a**); the center map shows the cumulative incidence rates (2007–2008) at the district level to allow comparison (**b**) and the school-based prevalence survey of 2007–2008 (**c**)

The second comparison, illustrated in Fig. 6, is at district level between the incidence-based map and two prevalence-based maps. The first (see Fig. 6a) is the prevalence map of 2008 published by WHO in 2010 [46]. The WHO map was produced using United Nations population data and prevalence estimations based on the procedure developed by Chitsulo et al. [21]. If we compare the WHO map with the one based on incidence data (Fig. 6b) there are obvious similarities, but with a notable exception for the Gicumbi district (red unit in Fig. 6a). According to the WHO mapping, Gicumbi district is hyper-endemic while in reality, the nationwide school-based prevalence survey (see Fig. 6c) identified three out of four sampled schools to be non-endemic (with a prevalence of 0%).

In order to obtain a reliable disease burden measure, a prevalence survey is always needed. But the use of incidence data is suitable to identify high-resolution spatial patterns of disease distribution at the national scale. Disease maps based upon incidence data might also be used to guide a spatially explicit sampling procedure for improved prevalence sampling. This is important given that the spatial representability of surveyed schools is not well elaborated in current WHO guidelines for the evaluation of helminthiasis at the community level [47]. In some case, the cross-sectional survey was influenced by accessibility by four-wheel drive car [48]. Inaccessible areas with poor quality or non-existent roads around valleys and perennial water bodies are usually poorly represented in school samples obtained, while those have now been identified as potential high-risk areas [49].

#### *S. mansoni* Incidence rates and environmental risk factors

This study detected a significant relationship between *S. mansoni* incidence rates and potential environmental risk factors as summarized in Table 3. Elevation and sand percentage in the soil are negatively correlated with *S. mansoni* incidence, whereas temperature, rainfall, and wetlands used for rice cultivation are positively associated. These findings are consistent with existing knowledge on the environmental conditions required for the development of the intermediate host snails and hence, can influence transmission risk. The related physical and ecological parameters were similar to other studies [1, 50, 51]. Low elevation, valley wetlands, warm and humid conditions and less sandy soil create favorable conditions for *S. mansoni*, as host snails inhabit places with lower altitudes and wetlands with natural or cultivated vegetation [50, 52]. The TSI offer additional and important information for discriminating the implications of those changes on wetness conditions more than soil types do [53].

Prior research has demonstrated that *S. mansoni* infection can increase as a result of the construction of dams or irrigation schemes [54]. Indeed, within irrigation systems transmission is focal and primarily due to localized contamination of habitats with human excreta containing Schistosoma eggs [55]. The newly identified hotspot in Nyagatare district (hotspot 3 in Fig. 5) is close to a more than 1000 ha irrigation scheme for rice cropping. Furthermore, intense cultivation such as flowers and sugarcane within Nyabarongo wetland can be linked with the hotspot identified in Kigali city (see hotspot 4 in Fig. 5). A similar situation was also reported for Minas Gerais State in Brazil [56]. This indicates that vulnerability to *S. mansoni* is not limited to rural populations, school children or women of childbearing age but extends to entire communities.

#### Limitations of this research approach

Although the incidence data from routine health records have a high quality as described in the methodology section, the number of confirmed cases will still only be a fraction of all infected persons and will mostly concern patients manifesting clinical symptoms. If the incidence database could be enriched with additional information (e.g. intensity of infection in tested stools, the total number of investigated subjects and their age and sex) the outcomes of the analysis would become even more robust.

Regarding the patterns analysis and clusters mapping, we used the Global Moran’s I and Local Gi* (d) statistic instead of local Moran’s I. However, both statistical tests are related; the correlations between nearby values of the statistics are derived and verified by simulation. In this specific case, with aggregated values at HFSA level, the spatial autocorrelation was weak (Moran’s I is not closer to +1) and statistically significant hot spots, cold spots, and spatial outliers were only distinguished with Local Gi* (d) statistics. Thus, the linear regression, although not the best approach for the spatially autocorrelated dataset, presented satisfactory results for exploratory analysis of disease risk factors. Given that there was no strong spatial dependency between closer values (as proven by semi - variogram test). Additionally, the risk for a fixed spatial pattern was prevented by sampling randomly the model calibration data subset.

Somewhat unexpected is that none of the demographic and socio-economic variables had a significant contribution to explaining *S. mansoni* incidence variability at HFSA level. A plausible explanation for this is that in Rwanda there is low variability in levels of school attendance, access to improved water sources, proper sanitation, and wearing of shoes [16, 17]. In Rwanda, conditions have much improved as a result of significant policy achievements in the last two decades [57, 58]. Consequently, additional information about people’s behavior would be required to further improve our understanding of *S. mansoni* risk factors. Possible examples are the habit of taking off shoes while working in the field, defecation in bushes when working on the land (and thus far from the home toilet), which may be important to better understand *S. mansoni* exposure [1, 9, 59].

The multiple regression models explain a lot of the observed spatial variability of *S. mansoni* incidence rates as a function of possible locational risk factors. The district model performed better than the model at the more detailed HFSA level. This is consistent with the fact that aggregation of data causes linearization contributing to overestimation in linear regressions [60].

## Conclusion

This study has demonstrated that in Rwanda prevalence and incidence data for *S. mansoni* are highly correlated. Given the availability of reported cases for each health facility in Rwanda, a high resolution spatially explicit statistical investigation of *S. mansoni* hotspots is feasible. The identified risk areas provide an appropriate basis to guide *S. mansoni* control programs at a much more detailed spatial scale than was possible before. In addition, the most important physical, ecological and climatic risk factors for *S. mansoni* transmission in Rwanda were identified. It was also shown that intensive agricultural use and transformation of wetlands for rice cultivation contributes to the spreading of *S. mansoni* into previously non-endemic areas. In line with environmental health impact monitoring and evaluation for wetland based development projects, a specific policy is required to address and reduce potential disease risk associated with rural development efforts. Finally, use of routinely collected incidence data opens the door for spatiotemporal analysis of *S. mansoni* and environmental risk factors which will vary in space and time.
